# How to Hit Mesenchymal Stromal Cells and Make the Tumor Microenvironment Immunostimulant Rather Than Immunosuppressive

**DOI:** 10.3389/fimmu.2018.00262

**Published:** 2018-02-19

**Authors:** Alessandro Poggi, Serena Varesano, Maria Raffaella Zocchi

**Affiliations:** ^1^Molecular Oncology and Angiogenesis Unit, Policlinico San Martino, Genoa, Italy; ^2^Division of Immunology, Transplants and Infectious Diseases, San Raffaele Scientific Institute, Milan, Italy

**Keywords:** mesenchymal stromal cells, carcinoma-associated fibroblast, tumor-associated fibroblast, tumor microenvironment, immunosuppression

## Abstract

Experimental evidence indicates that mesenchymal stromal cells (MSCs) may regulate tumor microenvironment (TME). It is conceivable that the interaction with MSC can influence neoplastic cell functional behavior, remodeling TME and generating a tumor cell niche that supports tissue neovascularization, tumor invasion and metastasization. In addition, MSC can release transforming growth factor-beta that is involved in the epithelial–mesenchymal transition of carcinoma cells; this transition is essential to give rise to aggressive tumor cells and favor cancer progression. Also, MSC can both affect the anti-tumor immune response and limit drug availability surrounding tumor cells, thus creating a sort of barrier. This mechanism, in principle, should limit tumor expansion but, on the contrary, often leads to the impairment of the immune system-mediated recognition of tumor cells. Furthermore, the cross-talk between MSC and anti-tumor lymphocytes of the innate and adaptive arms of the immune system strongly drives TME to become immunosuppressive. Indeed, MSC can trigger the generation of several types of regulatory cells which block immune response and eventually impair the elimination of tumor cells. Based on these considerations, it should be possible to favor the anti-tumor immune response acting on TME. First, we will review the molecular mechanisms involved in MSC-mediated regulation of immune response. Second, we will focus on the experimental data supporting that it is possible to convert TME from immunosuppressive to immunostimulant, specifically targeting MSC.

## Introduction

Mesenchymal stromal cells (MSCs) are a key component of solid tumor microenvironment (TME) (1–4). They include fibroblasts, myofibroblasts, pericytes, vascular or lymphatic endothelial cells, and undifferentiated mesenchymal stem cells. These cells produce the large part of the extracellular matrix and are involved in the homeostasis of tissues in different organs. There is experimental evidence that MSC can be influenced by tumor cells and, in turn, regulate tumor cell growth and expansion (1–4). In many instances, MSCs are driven by tumor cells to modify the extracellular matrix components, allowing tumor cell adaptation to the surrounding microenvironment and eventually metastasization (1–4). In healthy tissues, MSCs represent the network on which epithelial cells, blood, and lymphatic vasculature are organized and polarized. After receiving a danger signal, induced by biological, chemical, or physical injury, MSCs respond to maintain tissue homeostasis, favoring the repair of the tissue and reconstituting the healthy condition. During this process, MSCs come across the innate and adaptive arms of the immune system. This interaction should be highly regulated to avoid, on one hand, uncomplete repair and, on the other hand, an inefficient shut down of immune response leading to chronic inflammation (1–4). During this process, microenvironment is plenty of stimuli that, when out of control, can favor the overwhelming growth of epithelial cells with genetic alterations that are the basis of oncogenesis (1–4). Thus, the generation of a neoplasia can be dependent on the response of MSC to pathogenetic signals and to the cross-talk with immune and epithelial cells. Indeed, MSC can show immunosuppressive properties that are necessary during wound healing and repair process, but this feature is a drawback when a tumor is growing within the damaged tissue (1–4). Herein, we will briefly review the main features of MSC, from phenotype to functional properties, to clarify the molecular mechanisms whereby these cells can become immunosuppressive. Then, we will focus on the possible ways to modify MSC behavior and commute the TME from immunosuppressive to immunostimulant.

## MSC: Phenotypic and Functional Characteristics

**Figure 1:**
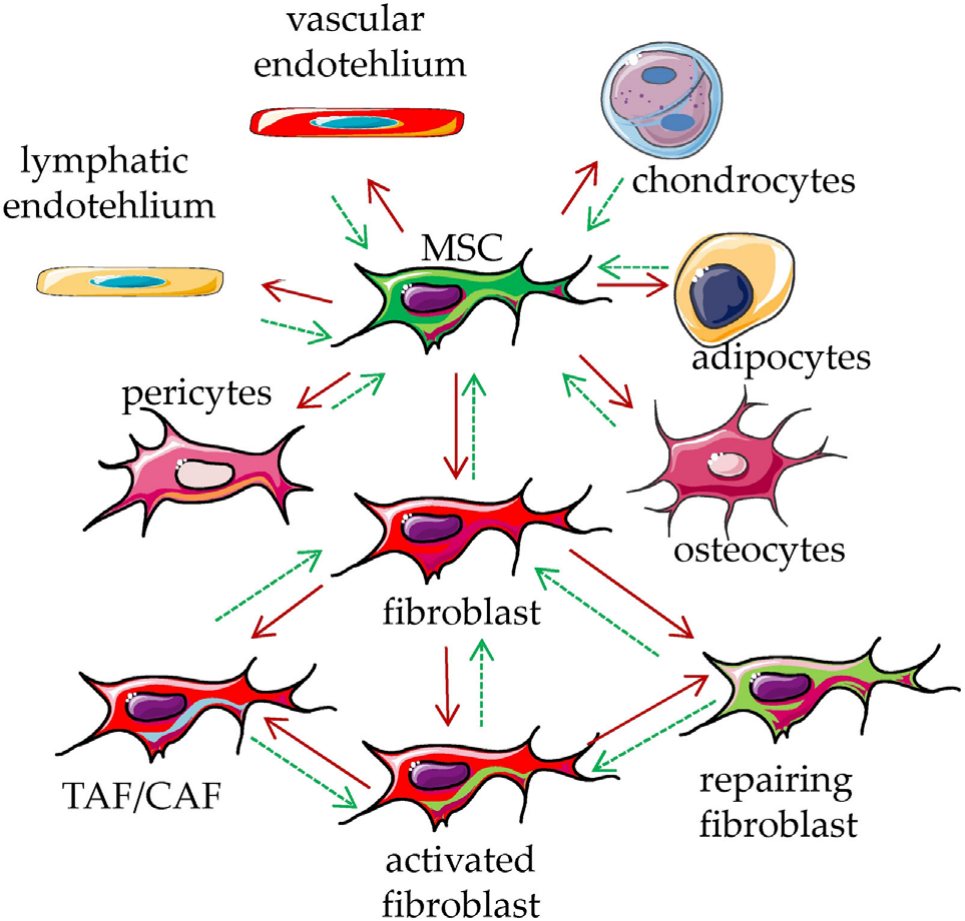
Mesenchymal stromal cell (MSC) plasticity. MSCs are present in every tissue, where they represent a key component characterized by the ability to differentiate into several types of mesodermal cells, including osteocytes, adipocytes, chondrocytes, endothelial cells, pericytes, and fibroblasts (red arrows). It is not clear whether all these kinds of cells can in turn de-differentiate back to MSC (green dotted arrows). The function of these cells is to maintain the homeostasis of the tissue/organ where they are present, regulating the production of the extracellular matrix components. Upon stimulation with physical, chemical, or biological stimuli, they participate in the reconstitution of the equilibrium among cellular and matrix components of a given tissue, leading to damage repair. They can be considered as sensor of the tissue conditions which can coordinate the molecular mechanisms that maintain tissue integrity. Upon influence of microenvironment, fibroblasts can lead to tumor-associated/carcinoma-associated fibroblasts (TAF/CAF), activated fibroblast and fibroblast involved in repair of the tissue.

To talk about a cell type and its functional features, it is important to define their phenotypic and functional characteristics to avoid confusion among the different reports found in the literature (1–4). To simplify, a very comprehensive definition of MSC is that they are cells of mesodermal origin that are neither epithelial cells nor leukocytes (1–4). The term “MSCs” have been coined by the Mesenchymal and Tissue Stem Cell Committee of the International Society for Cellular Therapy (5, 6). These “MSCs” are defined as multipotent mesenchymal cells that can be found in several different tissues (1–6) and can differentiate, under appropriate culture conditions, into adipocytes, osteoblasts, and chondrocytes (5–10). It has been shown that adipocytes and osteoblasts can be obtained from cultures of fibroblast-like cells from skin biopsies (11). Thus, it is possible that the cultures set up to select fibroblasts contain residual stem cells that in turn differentiate to other stromal cells, such as adipocytes, chondrocytes, and osteoblasts (5–11). This implies that MSC is not a synonym of mesenchymal stem cell. Also, MSC can include fibroblasts, endothelial cells, pericytes, and mesenchymal stem cells (1–4); in turn, mesenchymal stem cells are precursors of osteoblasts, chondrocytes, and adipocytes, which can be considered as MSC. On this basis, the different cell types can be distinguished for their differentiation potential and preferential production of a given component of extracellular matrix, related to the grade of differentiation (1, 3). It is not clear whether all these kinds of cells can de-differentiate to give rise to different members of MSC, in other words, what is the degree of plasticity of a differentiated MSC (Figure 1). It is conceivable that the tissue microenvironment of a given organ leads a stem cell to differentiate into a given MSC with peculiar functional properties (1–4). If this is the case, any kind of cell derived from mesenchymal stem cells should share some phenotypic and functional characteristics (Figure 1). Although several phenotypic characteristics and functional activities of MSC have been well reviewed recently (1–10), we will briefly summarize the most relevant phenotypes, found in MSC cultured *in vitro*, related to their function in TME.

Collectively, MSC can be identified as cells that grow adherent to plastic, with elongated-diamond (fibroblast-like) shape, expressing a definite set of markers, including CD73, CD90, and CD105, but lacking the typical hematopoietic lineage and non-lineage-specific markers, such as CD34, CD45, CD14, CD11b, CD31, CD79, CD19, and HLA-DR (2–4). In some instances, some MSC cultures show peculiar markers, such as the fibroblast activation protein (FAP) found in tumor-associated fibroblasts (1–3), but it is hard to identify subpopulations of MSC on the basis of the bimodal expression of a given antigen. In other words, it is difficult to define a MSC-specific marker, as occurs in the case of CD4^+^ or CD8^+^ lymphocytes. Indeed, although distinct fibroblast subpopulations have been reported, based on the different intensity of expression of some cell surface molecules (1–4), it is not easy to distinguish these markers by immunofluorescence. In addition, MSC can produce a variety of cytokines, chemokines, and factors, such as basic fibroblast growth factor, heparin epidermal growth factor, insulin-like growth factor (IGF) 1, keratinocyte growth factor, platelet-derived growth factor-β chain (PDGF-β), vascular endothelial growth factor (VEGF), and angiopoietins, involved in tissue repair (1–3). Indeed, the main function of MSC is thought to be the repair of injuries: this process is triggered by both differentiation of MSC in specialized tissue elements, producing peculiar extracellular matrix proteins, and regeneration of the tissue and vessel architecture (1–4). In this context, the immunosuppressive properties of MSC have been demonstrated for differentiated mesenchymal stem cells (12–15) and fibroblasts (11). Unfortunately, several MSC properties have been discovered after *ex vivo* expansion upon culture *in vitro*, so that the resulting cell population may represent a selected subset of MSC. This can also explain why findings reported from different laboratories may be conflicting (16, 17). Another relevant point to be considered is the culture ratio between MSC and tumor cells or leukocytes. Several reports have shown that the maximal inhibiting effect exerted by MSC on lymphocyte functions is achieved at MSC-lymphocyte ratios ranging from 1:1 to 1:10 (16–28). While it is possible that these ratios can be found also *in situ*, it is evident that in *ex vivo* conventional cultures the microenvironment does not dynamically change as it occurs *in vivo*. Indeed, in the large majority of reports, the time points chosen to analyze an inhibiting effect were set up after several days of co-culture (16–30). This implies that the vitro culture microenvironment is composed of metabolites and factors not necessarily present *in situ*; indeed, *in vivo*, blood and lymphatic vessels are involved in the clearance and renewal of the tissue milieu (31). Experimental evidence has been reported to support that MSC can display immunosuppressive behavior *in vivo* (32–38). However, a direct demonstration of the immunosuppression exerted by MSC is far from to be demonstrated and even the potential relevance of these cells for regenerative medicine is not unequivocally proven (32).

To summarize, MSCs are present in both healthy and neoplastic tissues as undifferentiated and differentiated cells that maintain the homeostasis with a strong relevance in regulating epithelial cells growth and immune response.

## MSC and Carcinoma-Associated Fibroblasts

Mesenchymal stromal cells present in solid tumors are fibroblasts that are called carcinoma (or tumor)-associated fibroblasts (CAF or TAF) (1–4). These cells display characteristics different from MSC of healthy tissues, conceivably related to the surrounding milieu (1–4). Several factors produced by MSC, such as hepatocyte growth factor (HGF), IGF1, and FGF, in TME can interact with surface receptors on tumor cells influencing their growth (1–4). In addition, pro-angiogenic factors, such as VEGF and PDGF, produced by MSC can favor tumor cell growth indirectly, promoting the tumor niche neovascularization (1–4). Thus, it is evident the possibility of blocking tumor cell growth by inhibiting the VEGF and/or the PDGF signaling axis (39–41). Of course, also tumor and immune cells, including tumor-associated macrophages and tumor-infiltrating lymphocytes (of both the innate and the adaptive arm of the immune system) can produce these factors; thus, the block of angiogenesis can hit several components of the TME, besides MSC. MSCs are also able to release TGF-β; this cytokine can exert several opposite effects on tumor cells, depending on the type and stage of tumor (42). Indeed, TGF-β can act as a tumor promoter as well as a tumor suppressor (42); furthermore, this cytokine is a relevant factor in epithelial–mesenchymal transition (EMT), a phase of tumor life which is considered essential for the generation of cancer metastasis (42). Recently, molecular mechanisms underlining the cross-talk between MSC and carcinoma cells have been deeply reviewed (1–4, 43–47). It is of note that, besides the direct MSC–tumor cell interactions, exosomes released by MSC can contain factors, such as micro RNA (47–56), that may drive either solid tumor cell apoptosis or tumor growth and spreading.

## MSC as Regulators of Immune Response

**Figure 2:**
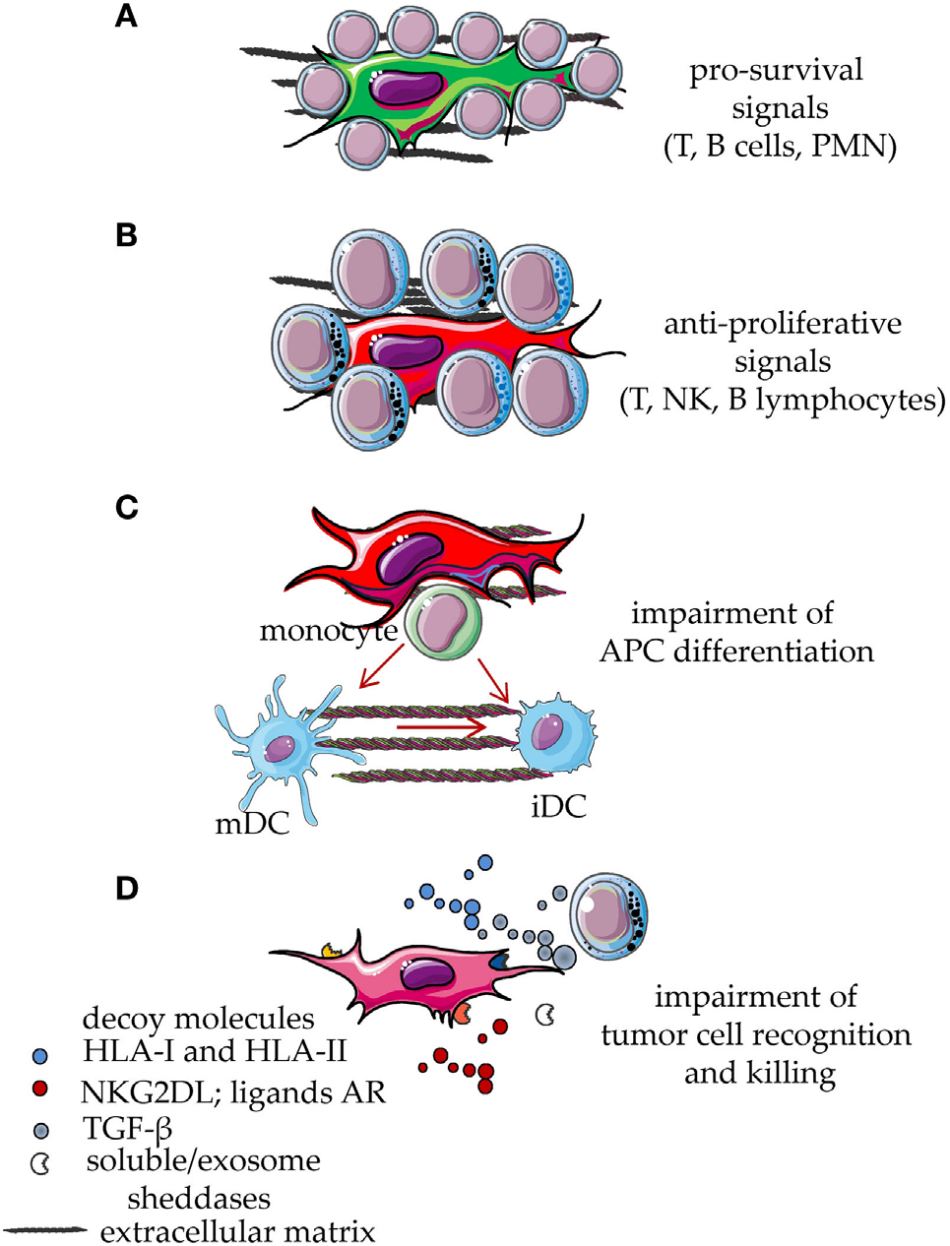
Functional behavior of mesenchymal stromal cells (MSCs) interacting with cells of the immune system. MSCs display several functional abilities during the interaction with cell of the immune system. Resting leukocytes are typically supported by MSC through direct cell-to-cell contact involving different types of receptor on the leukocyte membrane and the corresponding ligands expressed on MSC or on differentiated MSC. Some soluble factors and interleukins are also involved, such as stromal-derived factor 1, IL-6, and IL-15. Due to this interaction, leukocytes receive an anti-apoptotic signal which leads to their survival **(A)**. This effect is also involved in the maintenance of the neoplastic counterpart of T, B lymphocytes and myeloid cells. After signals inducing proliferation, cytokine release, or activation of cytolytic machinery, MSCs exert a potent inhibitory effect that reduces leukocyte proliferation and effector functions **(B)**. In addition, activated MSCs interfere with the differentiation of monocytes to immature DC (iDC) or mature DC (mDC), thus blocking the generation of professional antigen-presenting cells (APC) **(C)**. The release *via* microvescicles, exosomes or in soluble form, of decoy molecules such as HLA-I, ligands for NKG2D or other activating receptors involved in tumor cell recognition and killing, hampers the anti-tumor activity of T and NK lymphocytes **(D)**. Furthermore, MSC can release TGF-β, sheddases, such as metalloproteinases, and a disintegrin and metalloproteinase members, which can induce the release of decoy receptors from MSC, tumor cells, and bystander cells in the microenvironment. TGF-β can inhibit tumor cell recognition reducing the activation-induced increase of NKG2D expression on anti-tumor effector lymphocytes **(D)**. All these events eventually lead to the impairment of both innate and adaptive immune responses.

There is experimental evidence that MSC, mainly the MSC from bone marrow, can suppress immune responses *in vivo* (1–4, 10, 23, 24). In particular, the ability of MSC to reduce graft-versus-host disease (GVHD) has been reported (32–38). *In vitro* experiments have shed a light on which leukocyte populations MSC can regulate (1–4). MSC can act on both the innate arm and the adaptive arm of the immune system, blocking the expression and function of activating surface receptors on effector cells, impairing the maturation of antigen-presenting cells (APC) and favoring the expansion of regulatory cells (1–4, 12, 26, 57–67). This evidence derives from experiments where, in well-defined settings, different cells of the immune system are cocultured with a feeder layer of MSC and triggered by a given stimulus (12, 26, 68–72). Usually, such stimuli can induce proliferation, secretion of pro-inflammatory cytokines, or acquisition of a potent cytolytic potential. Upon coculture with MSC, both lymphocytes and APC are impaired in the acquisition of functional features essential to evoke a “normal” immune response (12, 26). Indeed, APC do not differentiate adequately to permit a full response to antigen-dependent or -independent stimuli (12, 26) and do not express high amounts of accessory molecules, such as CD80 and CD86, essential to deliver an optimal second signal. On the other hand, T lymphocytes express low levels of receptors, including CD25, typical of an activation state and do not respond to IL2 (12, 22, 23). The generation, in cocultures with MSC, of T cells with regulatory activities is an additional mean through which MSC can indirectly deliver an inhibiting signal to immune response (57, 58). Several papers have pointed out that different types of MSC can exert different degrees of inhibition of immune responses (1–4). In addition, differentiated MSC can still act as potent regulators of immunity (12, 72, 73). However, depending on the type of fully differentiated mesenchymal cells, pro-stimulating or pro-inhibiting effects have been described. For instance, it has been shown that mature adipocytes can trigger T cell proliferation and both HLA-DR and HLA-I appeared to be involved (74–76). Indeed, mature adipocytes express low levels of HLA-G, a surface structure responsible for the MSC-mediated T cell inhibition (76). It is of note that the ability of adipocytes to stimulate T cells was related with a stronger expression of HLA-DR and of the master transcriptional regulator CIITA factor, compared to de-differentiated adipocytes (76). On the other hand, fully differentiated chondrocytes can inhibit T cell proliferation triggered through the CD3–CD28 activating receptors, impairing CD25 expression. More importantly, chondrocytes can affect the differentiation of monocytes to dendritic cells (12). All these effects can in turn amplify each other, thus making the immunoregulatory activity of MSC really strong (Figure 2).

## Molecular Mechanisms of the Immunoregulation Mediated by MSC

**Figure 3:**
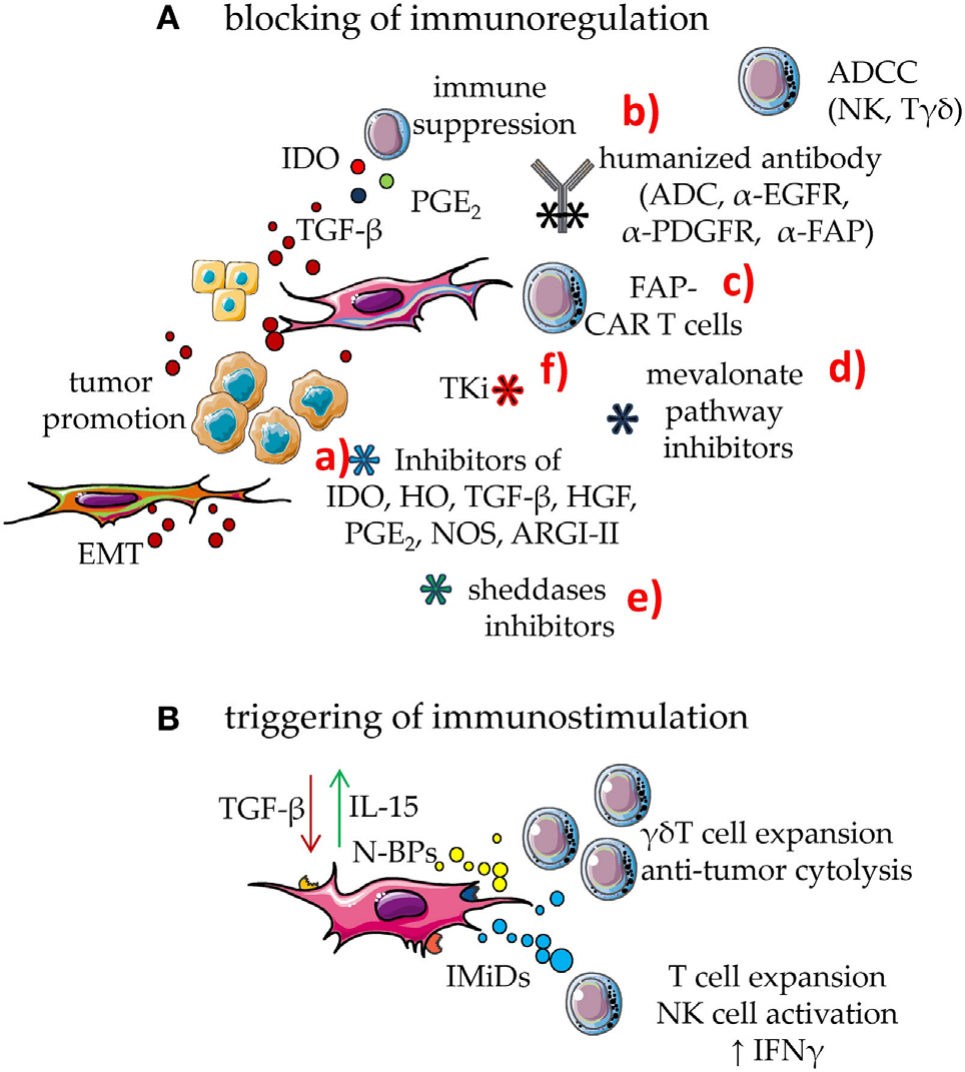
Means to enhance the immune response in the tumor microenvironment (TME). To counteract the mesenchymal stromal cell (MSC)-mediated downregulation of immune response, two main approaches can be utilized: **(A)** blocking of immunosuppressive effect; **(B)** triggering MSC to be immunostimulant rather than immunosuppressive. **(A)** MSC can downregulate immune response through several soluble factors such as indoleamine 2,3 dioxygenase (IDO) prostaglandin E_2_ and TGF-β. In turn, TGF-β from MSC, tumor cells, and bystander cells in TME can support tumor cell growth and dissemination. This latter event is linked to epithelial–mesenchymal transition (EMT) that triggers the generation of metastasis. The blockade of MSC immunosuppression can be obtained by several means: (a) drugs that inhibit the activity or the generation of molecules involved in immunosuppression such as inhibitors of IDO, HO, TGF-β, hepatocyte growth factor (HGF), PGE_2_, NOS, and ARGI–II; (b) antibodies directed either to MSC growth receptors, as the epidermal growth factor and platelet-derived growth factor (PDGF) or to the fibroblast activation protein (FAP). It is of note that some of these receptors are shared by tumor cells; thus, human or humanized antibodies-based therapy can target both MSC and cancer cells. These antibodies act inhibiting the effect of a given growth factor but also impairing the function of the target molecule. In addition, they trigger complement-dependent cytotoxicity and antibody-dependent cellular cytotoxicity (ADCC) elicited by Fcγ receptor-expressing cells, including natural killer (NK) cells and γδ T cells. These antibodies can be a portion of antibody–drug conjugates (ADC), which join the antibody-mediated effect to that of a cytotoxic drug, leading to a strong inhibition of tumor cell growth or MSC-mediated functions. (c) cytotoxic T cells equipped with chimeric antigen receptors (CARs) specific for FAP (FAP-CAR T cells) that can recognize FAP^+^ cells; (d) drugs affecting the mevalonate pathway that is essential for both MSC and tumor cell metabolism; unfortunately, mevalonate is relevant also for the development of an optimal immune response; they should therefore be used carefully; (e) inhibitors of sheddases, as matrix metalloproteinase and a disintegrin and metalloproteinases, which can inhibit tumor cell growth limiting the generation of growth factors in a suitable form to trigger proliferation; furthermore, these inhibitors should impair the generation of decoy molecules, reducing the competition between membrane and soluble ligands for activating receptors on effector lymphocytes; (f) tyrosine kinase inhibitors (TKi) which block the activity of MSC besides hindering tumor cell growth. **(B)** Immunomodulatory drugs (IMiDs), among which thalidomide, pomalidomide, lenalidomide, and avadomide can trigger the innate and the adaptive immune responses, besides hampering angiogenesis in the tumor. Aminobiphosphonates (N-BPs), such as zoledronic acid, can interfere with the mevalonate pathway strongly enhancing the production of isopentenyl pyrophosphate (IPP) and dimethyl allyl pyrophosphate (DMPP). These small pyrophosphates can trigger the expansion of γδ T cells of the Vδ2 subset, a cell population with potent anti-tumoral capabilities. Furthermore, Vδ2^+^ T cells express the FcγR involved in ADCC, reinforcing the anti-tumor effect of human/humanized antibodies.

Mesenchymal stromal cells regulate immune response by different means (1–4), shared with other components of the TME, such as myeloid-derived suppressor cells (MDSC), tumor cells, and infiltrating Treg lymphocytes (1–4, 77–84). Indeed, indoleamine 2,3 dioxygenase (IDO), hemeoxygenase (HO), arginase 1 and 2 (ARG1 and ARG2), nitric oxidase synthase 2 (NOS2), HGF, TGF-β, IL10, prostaglandin E_2_ (PGE_2_), and adenosine are all factors involved in the MSC-mediated regulation of innate and adaptive immunity (1–4, 23, 85–92) (Figure 3). It is of note that several of these factors are upregulated by inflammatory stimuli, such as IFN-γ (69). IDO and PGE_2_, are strongly induced upon inflammation, conceivably to switch off the inflammatory response to danger signals. In the TME, IDO- and PGE_2_-mediated immunosuppression can be the marker of a physiological response triggered to favor tissue repair, but undesired because it favors also tumor cell growth. Indeed, IDO induces kynurenine synthesis that can strongly inhibit both the innate and the adaptive immune response (93–98). Furthermore, TGF-β is not only relevant for tumor cell growth but can also directly inhibit the function of anti-tumor effector cells. This cytokine downregulates, at the surface of natural killer (NK) cells, CD8^+^ cytolytic T cells and γδ T cells, the expression of the NKG2D activating receptor, which in turn cannot interact with the NKG2D ligands expressed by tumor cells. These events would limit the immunosurveillance to stress signals mediated by the growing tumor (1–4, 25). In addition, TGF-β is a critical factor to generate conventional CD4^+^CD25^high^^+^ Treg and regulatory γδ T cells (42, 99–104). Moreover, TAF expressing α-smooth muscle actin (SMA) can convert arginine in ornithine through the involvement of ARG2; this leads to the inhibition of TIL functional activities, especially in hypoxic conditions (88). PGE_2_ derived from NK–MSC cocultures can impair the IL-2-dependent upregulation of activating NK-cell receptors, such as members of the natural cytotoxicity receptors and DNAM-1, thus inhibiting melanoma cell recognition (20). Adenosine is an additional factor involved in MSC-mediated immunosuppression. Indeed, the ecto-5′-nucleotidase activity of CD73 expressed on MSC can catalyze the hydrolysis of the extracellular adenosine monophosphate (AMP) to adenosine. This metabolite can influence the activity of adenylyl cyclase, the synthesis of cyclic AMP and the function of PKA exerting potent immunosuppressive effects (90–92).

## Targeting MSC with Anti-Tumor Drugs

Tyrosine kinase inhibitors (TKi) are recent drugs that block the signaling cascade that follows the interaction of a growth factor with its specific receptor (105–107). It is not surprising that some TKi can affect MSC as well (Figure 3). Indeed, MSCs bear at the cell surface several receptors that can be considered as targets for tumor cell therapy with TKi. In particular, the expression on MSC of PDGFR-β and EGFR is well established; the effects of TKi such as imatinib, nilotinib, or gefitinib *in vitro* have pointed out that these drugs can affect both MSC proliferation and differentiation (108–122). These effects have been recently reviewed in very detail (122). It is clear from all these findings that, as expected, TKi can exert a strong inhibition on MSC growth and function, but their effects on MSC-mediated immunosuppression have not been studied. It is conceivable that the inhibition of MSC proliferation leads to the inhibition of MSC responsiveness to TME signals, but this is not determined yet. However, it has been recently shown that the encapsulated TKi sunitinib can work synergistically with vaccine therapy in an advanced mouse melanoma model, leading to the remodeling of TAF, collagen, and vessels of the tumor. Furthermore, TKi can induce a shift from Th2 to Th1 pattern of TIL, accompanied by an increment of these lymphocytes and a decrease of MDSC (123).

## Targeting MSC Antigens to Modulate TME

It is now evident that the immune system can have a significant role in limiting and controlling tumor cell growth (124–131). Indeed, both adoptive and immune check point inhibitor immunotherapies are based on the possibility of triggering, either passively or actively, the specific anti-tumor immune response (124–131). A third possibility of adoptive immunotherapy is the administration of tumor vaccines; however, tumor vaccination has led to contrasting results in clinical practice (132–138). In this setting, it is attractive to target not only tumor cells but also different components of the TME (40, 41, 139–162). Indeed, specific vaccines to tumor endothelial cells or blockers of the VEGF signaling have been used in preclinical studies, and clinical trials are ongoing (40, 41). MSC can become a target for anti-tumor vaccines as well (141–162). For instance, the strong production of collagen type I by MSC can interfere with the uptake of anti-tumor drugs (149, 150); thus the targeting of MSC and the inhibition of extracellular matrix components can render more sensitive tumor cells to chemotherapy. Furthermore, antigens shared by tumor cells and TAF can be good targets for a vaccine. The fibroblast activation protein (FAP), a member of the serine protease family, can be expressed by TAF at higher levels than on resident fibroblast of healthy tissue. In addition, FAP can be also expressed by tumor cells; this would imply that an immune-based therapy focused on FAP can beat both tumor cells and TAF (140, 143–162). Indeed, it has been shown, in a murine model, that FAP^+^ tumor cells can be used as a vaccine, leading to reduced vascular dissemination and elimination of different tumors. In the same model, tumor-infiltrating CD8^+^ T cells increased and a net decrease of intratumor TAF, accompanied by a reduced recruitment of cells with immunosuppressive phenotype, was found in treated animals (144). In this context, the use of the humanized anti-FAP monoclonal antibody sibrotuzumab has been proposed in non-small cell lung and colorectal cancer (CRC), but the pilot study in CRC did not reach the minimal requirements for the continuation of the trial (163–166). However, FAP has been considered as a target for redirected T cells or chimeric antigen receptor (CAR) T cells (158, 159, 162) (Figure 3). It has been reported that transfer of murine T cells transduced with FAP-CAR construct can affect tumor cell growth increasing the CD8^+^ T cell response. Also, the administration of anti-fibrotic agents, in several murine tumor models (E-G7 lymphoma, LLC1 Lewis lung cancer, or B16F1 melanoma) induced a strong increment of CD8^+^ T cells, NK activity, and humoral immunity and a sharp decrease of MDSC, Treg, stromal-derived factor 1, TGF-β, and PGE_2_ (162).

## Can MSC Counteract Cancer Development and Growth?

Taking together the findings reported, it appears clear that MSC as TAF should be a mean by which tumor cells are facilitated in their growth and spreading. Thus, the higher is the content of TAF in a given tumor, the faster will be the expansion of that tumor. TAF elimination leads to an enhancement of immune response and, at the same time, to a lower support of tumor cell growth. By contrast, recent evidence in pancreatic ductal adenocarcinoma (PDAC) indicates, that the depletion of αSMA^+^ myofibroblast, in a murine model can trigger tumor cell expansion and paradoxically accelerate disease progression (167). In addition, this depletion led to an increment of regulatory T cells without affecting NK cell infiltration. This was accompanied by a strong remodeling of the extracellular matrix composition and the therapy with CTLA-4 immune check point inhibitors could rescue the detrimental effect due to myofibroblast depletion. Furthermore, it appeared that the lower was the number of αSMA^+^ myofibroblast in human PDAC, the worse was the prognosis of patients (167). How to explain this unexpected effect? The simplest explanation is that the reaction due to αSMA^+^ myofibroblast represents a tool by which healthy MSC try to repair tissue and limit the expansion of PDAC, as suggested for other malign tumors (168–183). This phenomenon is known as desmoplastic reaction, which serves to repair tissue injury (175–182). It is conceivable that, at the onset of tumor growth, fibroblasts may function also as a physical barrier to tumor expansion. During tumor growth, due to the presence of subclones and/or cancer stem cells, this barrier can be modified by reciprocal cross-talk between tumor components and MSC. An additional explanation is that within αSMA^+^ myofibroblast are present subsets of cells with different functional behaviors, with either positive or negative effects on tumor cell growth. After depletion of all αSMA^+^ myofibroblast, these populations are lost and PDAC can grow without any brake (168, 173, 175, 177, 183). In such TME, immune system can receive misleading information with conflicting, undesired outcomes. Recently, it has been shown that NK cells can recognize and eliminate pancreatic stellate cells, *bona fide* myofibroblasts (171); this would suggest that innate immunity, in this case, can favor rather than inhibit tumor cell expansion by limiting stromal reaction.

## Research Gaps and Future Developments

At present, targeting MSC is complicated by the fact that a specific marker of these cells is missing (1–4). Indeed, MSCs have the property to differentiate and it is not clear whether there is also an intrinsic de-differentiation potential (1–4); these functional/plastic properties can impair the efficacy of a drug specific for a given MSC subpopulation. In addition, from data obtained in PDAC, it is clear that MSC can aid the host against cancer evolution. Finally, MSCs are present in each tissue and represent the key cell involved in the maintenance of the structural architecture of the whole body. Thus, therapeutic targeting of MSC should be made very carefully.

### Targeting MSC with Antibodies

All the above reported matters render the targeting of MSC not as specific as desired and possibly accompanied by relevant drawbacks. By contrast, tumor cell targeting can be more specific, since the marker used as target is more expressed in tumor cells than in their healthy counterpart. For instance, in Hodgkin lymphoma and non-Hodgkin lymphomas (NHL), tumor targeting can be really efficient (184–187). Indeed, in these instances, administration of therapeutic antibodies to CD30 or CD20 molecules can spare the healthy counterpart of B cells, because the target antigen is not expressed or is expressed at low levels. Also, B lymphocyte precursors can substitute the bystander healthy B cells damaged by target therapy (184–187). An additional relevant question is whether therapies aimed to eliminate cancer cells have also an effect on MSC. Indeed, humanized monoclonal antibodies (huAb), directed to receptors involved in the proliferation of tumor cells, including EGFR or Her2b, may hit MSCs that share these molecules at the cell surface (Figure 3). MSC targeting might be useful, on the one hand, but the availability of the therapeutic huAb can be reduced. Moreover, it is conceivable that anti-EGFR and/or anti-HerB2 huAb can affect MSC-tumor cell cross-talk due to the signal delivered upon huAb/receptor interaction (188, 189). The study of this interaction can shed new light on the reported unexpected effects observed with huAb therapy in some type of cancers, among which is CRC (190–193). As reported above, targeting FAP^+^ TAF, or αSMA^+^ myofibroblast has elicited unexpected drawbacks, since these cells can also function as negative regulators of cancer cell growth (167). The definition of subsets of MSC, myofibroblasts and even TAF, using a specific marker is a prerequisite to selectively hit the population that can favor the tumor cell growth and inhibit anti-tumor immune cell response. In this context, besides FAP, CD73, and CD105 (90, 91, 141–162), the finding that fibroblasts present in scar tissue and basal cell carcinoma express gremlin1, the secreted bone morphogenetic protein antagonist, would suggest that this can be a specific molecular target to distinguish TAF from healthy fibroblasts (194).

### Interference with EMT and Role of MSC

It is well known that EMT is a key step of the spreading of cancer cells far from the primary tumor (100, 101, 104, 195) (Figure 3). TGF-β plays a relevant role in EMT (101, 104, 194, 196–204); thus, it is conceivable that the blockade of TGF-β production by MSC can impair EMT (197). Some evidence is reported on the prometastatic effect of CAF in different types of cancer (198, 199, 202, 203). It is of note that EMT can be also triggered by anti-EGFR huAb therapy in squamous cell carcinoma of the head and neck. Indeed, it has been reported that cetuximab therapy can induce modifications in the expression of genes and proteins implicated both in EMT and in the extracellular matrix production by CAF (201). Importantly, upregulation of CXCL12, ASPN, and OLFM3, factors secreted by CAF, has been observed; CXCL12, through the interaction with its receptor CXCR4, can lead to CXCL12 and TGFβ production and concur to myelofibrosis (205). One can speculate that EGFR signaling can drive TME to generate therapy resistance involving CAF. Targeting CAF to reduce production and release of TGFβ, CXCL12, and matrix metalloproteinases (MPP) can limit cancer cell spreading favored by TGFβ and MPP and the anti-apoptotic effect of CXCL12 on tumor cells. Unfortunately, the clinical use of inhibitors of TGFβ and MPP is far from to be well established, although the interference with CXCR4/CXCL12 axis, using AMD3100 or huAb, is already applied in several clinical trials (204–210).

### Targeting Immunosuppressive Molecular Mechanisms of MSC with Inhibitory Drugs

The interference with MSC-mediated immunosuppressive molecular mechanisms, obtained using specific inhibitory drugs, is an additional mean by which the immune escape favored by tumor MSC can be avoided (1–4). In this context, all the inhibitors already used in therapeutic schemes to block IDO, HO, ARGI and II, NOS2, PGE_2_, and TGF-β activity can be employed to reduce MSC influence on tumor cell growth (211–217) (Figure 3). In this context, the immune check point inhibitors anti-PD1 and/or PDL-1 huAb can have an important role (132, 137, 218–220). Indeed, it has been shown that PD1 is involved in MSC immunoregulation of T and B cell proliferation (18, 221, 222). The striking therapeutic effect observed upon blockade of PD1–PDL-1 with huAb can be dependent not only on the direct effect on tumor cell–effector lymphocyte interaction, but also on the switch off of the inhibiting signal elicited by PD1–PDL-1 binding during lymphocyte–MSC interaction. PDL-1 expression is upregulated on MSC by IFNγ and this cytokine can upregulate IDO as well (223); this suggests that the combination of IDO and immune check point inhibitors can concur to overcome TME immunosuppression (224). Some drugs, such as hydroxy-methyl-glutaryl-coenzyme A (HMG-CoA) reductase inhibitors, can influence both immunosuppressive effects and cancer pro-survival signals delivered by MSC (28, 225) (Figure 3). Furthermore, it is clear that mevalonate, the metabolic product of the HMG-CoA reductase activity, is a key molecule for tumor cell fate (226). However, limiting mevalonate production can influence the functional behavior of macrophages and lead to regulatory T cell expansion, thus favoring tumor cell spreading (227). In addition, anti-tumor effector cell-mediated lytic activity is strongly reduced by HMG-CoA reductase inhibition (228–232). This can be related to the decrease of cholesterol content in lymphocyte membrane that limits the formation of rafts; these rafts are essential in the delivery of the activating signals that lead to granzyme and perforin release, upon effector–target interaction (232, 233), and consequent target cell killing. Thus, it is relevant to design inhibitors of mevalonate pathway that can be delivered specifically to MSC in order to limit tumor cell growth sparing immune surveillance.

### Drugs to Transform MSC from Immunosuppressive to Immunostimulant

Another approach to downregulate the inhibitory effect of MSC on immune system is to convert their behavior from immunosuppressive to immunostimulant. Recently, it has been demonstrated, both in NHL and CRC, that priming of MSC, derived from lymph nodes or colon mucosa, with the aminobisphosphonate (N-BP) zoledronic acid can trigger Vδ2 T cell proliferation (25, 234, 235). In NHL, zoledronate-pulsed MSC are impaired in the secretion of TGF-β, whereas there is an increment in the production of IL-15 (234) (N-BPs in Figure 3). It should be defined whether priming with zoledronate can favor the expansion of other anti-tumor effector cells that are inhibited by MSC and whether MSC can become a target of Vδ2 T cells. If this is the case, the specific delivery of zoledronic acid to the lymph node TME would trigger anti-tumor immunity. It is well known that N-BPs have a strong tropism to bone (236); for this reason they are commonly used to treat neoplasias primarily localized in the bone, such as multiple myeloma, or bone metastases of different carcinomas (237–239). In these instances, N-BPs have a dual effect: support the deposition of bone matrix to repair the osteolytic damage induced by tumor cells and trigger γδ T cell-mediated anti-tumor immune response (237–241). When tumors are localized in other tissues, a major issue for the administration of N-BPs is to efficiently target the tumor outside the bone. It can be hypothesized that the generation of antibody–drug conjugates (ADC) (242), made of huAb linked to N-BPs, can be a good tool to deliver N-BPs to a specific tumor site. So far, ADC have been developed with huAb specific for a tumor marker linked to cytotoxic drugs, the specificity of the antibody being the key parameter to maximize anti-tumor effect. It is conceivable that also the linkage of immunostimulant drugs to huAb specific for tumor cells and MSC can combine the specifity for the target with the triggering of anti-tumor γδ T cell immune response.

Immunomodulatory drugs (IMiDs), from the first described thalidomide to the recent reported avadomide (CC-122) (Figure 3), can affect both directly and indirectly tumor cell growth (243–251). Indeed, it has been reported that IMiDs can impair cereblon, a ubiquitin ligase constitutive in every cell type but crucial for cancer cell survival, causing mis-regulation of developmental signaling molecules and generation of reactive oxygen species, which in turn kill tumor cells. Furthermore, IMiDs inhibit tumor neoangiogenesis leading to the reduction of tumor cell growth. IMiDs can also modulate NK cell number and function, besides co-stimulate T cell proliferation; these effects have led to their use in multiple myeloma and several types of lymphomas. IMiDs administration has been proven to be effective in clinical trials, because these compounds can hit different components of the TME, including MSC (124, 139, 239, 243–248). It is conceivable that a progressively larger application to several kinds of solid tumors, since these drugs have shown remarkable effects in CRC and sarcomas (249–272). Importantly, in the bone marrow microenvironment, IMiDs inhibit the production of IL-6, essential for myeloma cell growth, by regulating SOCS1 (273). In addition, these compounds affect osteoblast differentiation, indicating that bone anabolic therapeutics are needed in myeloma to counteract the negative effect on bone metabolism of IMiD exposure. In this instance, the use of N-BPs can favor the deposition of bone matrix, thus limiting the damage induced by IMiDs.

### Drugs to Interfere with the Generation of Decoy Receptors from MSC and Tumor Cells

MSC can release the MHC-class-I related molecules MIC-A, MIC-B, and the UL16-binding proteins (ULBPs) into TME, through the enzymatic activity of members of the a disintegrin and metalloproteinases (ADAMs) family (274–279). These released NKG2D-L can function as decoy ligands blocking the NKG2D-mediated recognition of cancer cells that usually express them on the cell membrane (277–281) (Figure 3). It is reasonable that ADAM10 and ADAM17 in MSC can act on such stressed molecules expressed not only by MSC but also by other cells present in TME. In addition, ADAMs can be released in exosomes and microvescicles by MSC, thus spreading their enzymatic activity. This would imply that ADAMs inhibitors can reduce the MSC-mediated release of stress molecules, allowing cancer cell recognition by immune cells and eventually leading to an increment of tumor cell killing (280). In this context, it is becoming evident that the analysis of MSC secretome is highly relevant to understand the physiological and pathological behavior of these cells (282). The targeting of ADAMs inhibitors to TME could be achieved again, using ADC which recognize MSC and/or tumor cells. Importantly, the delivery to MSC of drugs, such as N-BPs and ADAMs inhibitors, either alone or in combination with huAb as ADC, can take advantage of nanotechnology (283–285). Nanovectors can be artificially built with different morphology and physico-chemical properties (283–285). The choice of these parameters is relevant to design the optimal combination and obtain the maximal effect (283–285).

### The New Frontier of Three-Dimensional (3D) Models: To Study the Interactions among MSC, Tumor Cell, and the Immune System

The study of the functional cross-talk among MSC, tumor cells, and the immune system can be more reliable using 3D models instead of classical *in vitro* culture systems (272, 286–299). Indeed, in these 3D models, the control of cell culture conditions and the regulation of biomechanical stimuli can give relevant insight on how biophysical cues can influence stromal cell phenotype and function; this can clarify how these modifications impact on tumor drug sensitivity. In addition, the cross-talk of tumor and stromal cells with immune cells can be studied in detail, varying the experimental conditions in a setting that reproduces tissue architecture; this can spare time, limit the costs of animal experimentation and reduce the environmental impact of animal breeding farms (290, 295, 297, 299). These culture systems, validated by the EU Reference Laboratories (EURL-ECVAM) as preclinical models, are reproducible 3D culture microenvironments useful for studying pharmaceuticals or biological pathways (300, 301). Among them, the hydrogels of matrix components, such as collagen, fibronectin, or cell derivates such as Matrigel or amorphic scaffold have been used (286, 293, 295). More recently, in multiple myeloma a model that recapitulates the interactions among MSC, myeloma cells, endothelial cells, and bone remodeling has been set up in order to analyze dynamically the cross-talk among all these cell populations (273). Indeed, this 3D model uses silk protein-based scaffolds that allow active cell attachment and growth on the scaffolds, rather than passive encapsulation in 3D hydrogel cultures. This represents a unique model to analyze under mechanical stress, similar to the bone tissue, the interactions of cancer cells and bone in a 3D microenvironment. The interaction among tumor cells, anti-tumor lymphocytes and MSC can be achieved in different 3D experimental setting as tumor spheroids, organoids and 3D on-chip cell cultures (291, 297, 301, 302). The 3D models where metabolic microenvironment is dynamically changed are essential to confirm the findings obtained in the murine system regarding the role of PDL-1 blocking in tumor metabolism (303, 304). Infact, in a mouse sarcoma model, it has been shown that glucose consumption by tumors can metabollically inhibit T cell responses, impairing glycolytic activity and IFNγ production. More importantly, anti-PDL-1 antibodies can block tumor glucose utilization favoring T cell glycolysis and IFNγ release (304). To validate these findings and further analyze the mechanisms of regulation of metabolism of immune cells humanized mice can be employed (305). However, these mice are engrafted with human hematopoietic stem cells and, for this reason, should be immunodeficient. Although this model can aid in mimicking the pathophysiological conditions of human beings, it is evident that the large majority of TME is composed of murine cells. On the contrary, organoids of tumors from patients’ specimens can be obtained and analyzed in detail (306–309). For instance, it has been recently shown that human intestinal organoids can be generated and used, not only for research purposes but even to treat intestinal injury (310). In addition, bioprinting techniques have led to the biofabrication of accurate models that can recreate the biophysical and biochemical characteristics of a given tissue (292). Thus, in the near future, the cross-talk among the different components of the TME will be analyzed using more and more precise 3D models and organoids from a given patient to test the sensitivity to selected targeted therapy (289, 294, 302, 306, 311–313).

## Concluding Remarks

It is now clear that MSC represent a key player in regulating TME through direct cell-to-cell interactions, producing several cytokines and releasing exosomes (314–322). The secretome of MSC can play an important role in immunosuppression (319, 320): its modification with drugs can represent a new tool for drug delivery and cell-free regeneration after tumor injury (314–318, 321, 322). Because of the lack of specific markers that identify subsets of MSC, the specific targeting of these cells appears to be difficult, to achieve selective inhibition of immunosuppression. Furthermore, it is still to be elucidated whether different subsets of MSC, due to their plasticity, can represent functional subsets of cancer-associated fibroblasts (323–328). This would imply that a specific marker for the immunosuppressive MSC will be still elusive for a long time. Nevertheless, it is conceivable that drug combination therapies of cancer, which limit, on the one hand, tumor cell proliferation and, on the other hand, trigger immune responses, which already involve MSC. The *in situ* analysis of MSC functional features, together with their study in 3D tumor culture systems, would allow to clarify the existence in humans of MSC subsets and to assess the effects of drug treatment in order to choose the right combination of therapeutic means for each patient.

## Author Contributions

AP, SV, and MZ wrote, edited, and revised the paper. AP takes primary responsibility of the manuscript content.

## Conflict of Interest Statement

The authors declare no conflict of interest and the founding sponsors had no role in the design of the study; in the collection, analyses, or interpretation of data; in the writing of the manuscript, and in the decision to publish the results.
